# Phage-mediated virulence loss and antimicrobial susceptibility in carbapenem-resistant *Klebsiella pneumoniae*

**DOI:** 10.1128/mbio.02957-24

**Published:** 2024-12-23

**Authors:** Yanshuang Yu, Mengzhu Wang, Liuying Ju, Minchun Li, Mengshi Zhao, Hui Deng, Christopher Rensing, Qiu E. Yang, Shungui Zhou

**Affiliations:** 1 College of Resources and Environment, Fujian Agriculture and Forestry University, Fuzhou, China; 2 Fujian Key Laboratory of Traditional Chinese Veterinary Medicine and Animal Health, College of Animal Sciences, Fujian Agriculture and Forestry University, Fuzhou, China; University of Illinois Chicago, Chicago, Illinois, USA

**Keywords:** antibiotic resistance, bacteriophage therapy, *Klebsiella pneumoniae*, phage training, coevolution

## Abstract

Bacteriophages, known for their ability to kill bacteria, are hampered in their effectiveness because bacteria are able to rapidly develop resistance, thereby posing a significant challenge for the efficacy of phage therapy. The impact of evolutionary trajectories on the long-term success of phage therapy remains largely unclear. Herein, we conducted evolutionary experiments, genomic analysis, and CRISPR-mediated gene editing, to illustrate the evolutionary trajectory occurring between phages and their hosts. Our results illustrate the ongoing “arms race” between a lytic phage and its host, a carbapenem-resistant *Klebsiella pneumoniae* clinical strain Kp2092, suggesting their respective evolutionary adaptations that shape the efficacy of phage therapy. Specifically, Kp2092 rapidly developed resistance to phages through mutations in a key phage receptor (*galU*) and bacterial membrane defenses such as LPS synthesis, however, this evolution coincides with unexpected benefits. Evolved bacterial clones not only exhibited increased sensitivity to clinically important antibiotics but also displayed a loss of virulence in an *in-vivo* model. In contrast, phages evolved under the selection pressure against Kp2092 mutants and exhibited enhanced bacterial killing potency, targeting mutations in phage tail proteins gp12 and gp17. These parallel evolutionary trajectories suggest a common genetic mechanism driving adaptation, ultimately favoring the efficacy of phage therapy. Overall, our findings highlight the potential of phages not only as agents for combating bacterial resistance, but also a driver of evolution outcomes that could lead to more favorable clinical outcomes in the treatment of multidrug resistance pathogens.

**IMPORTANCE:**

Carbapenem-resistant *Klebsiella pneumoniae* represents one of the leading pathogens for infectious diseases. With traditional antibiotics often being ineffective, phage therapy has emerged as a promising alternative. However, phage predation imposes a strong evolutionary pressure on the rapid evolution of bacteria, challenging treatment efficacy. Our findings illustrate how co-evolution enhances phage lytic capabilities through accumulated mutations in the tail proteins gp12 and gp17, while simultaneously reducing bacterial virulence and antibiotic resistance. These insights advance our understanding of phage-host interactions in clinical settings, potentially inspiring new approaches akin to an “arms race” model to combat multidrug-resistant crises effectively.

## INTRODUCTION

The global rise of antimicrobial resistance (AMR) and its associated morbidity from bacterial infection has been recognized as a critical global health crisis (1). In 2019 alone, multidrug-resistant (MDR) infections were responsible for an estimated 1.27 million deaths worldwide, with particular concern attributed to carbapenem-resistant *Klebsiella pneumoniae* (CRKp), being one of the most serious Gram-negative pathogens prioritized by the World Health Organization (2, 3). CRKp strains were shown to harbor resistance genes such as *bla*_KPC-2_ and *bla*_NDM-1_, enabling them to hydrolyze nearly all β-lactam antibiotics, rendering CRKp infections virtually untreatable (4). Although antibiotics remain in their key role when treating and preventing bacterial infections, their declining effectiveness has made it urgent to seek alternative antimicrobial strategies.

One promising alternative is the use of bacteriophages (phages), a natural killer of bacterial cells. Phages display several advantages over traditional antibiotics, including (i) the vast biodiversity of phages provides a rich source for therapeutic applications (5, 6); (ii) lytic phages are able to self-replicate within bacterial hosts, facilitating rapid amplification (7); and (iii) phages operate independently of antibiotic-resistance mechanisms, presenting a distinct mode of action against bacterial pathogens (5, 7). Successful cases of phage therapy overcoming antibiotic-resistant infection, such as inflammatory bowel disease (8), *Acinetobacter baumannii* infections (9), and *Salmonella* colonization (10), have suggested its potential as a remedy for difficult-to-treat MDR infections (11, 12). However, similar to antibiotics, the efficacy of phage therapy is challenged by the evolutionary arms race between phage and bacteria, leading to the emergence of phage resistance (13, 14).

The co-evolutionary dynamics occurring between phages and bacteria, spanning billions of years, have significantly shaped microbial ecology and evolution (15). Phage infections mainly drive bacterial evolution through mutations in phage receptors and the activation of defense mechanisms such as restriction-modification (16) and abortive infection systems (17). In turn, phages have developed counter-defense strategies to co-exist within bacterial populations, including gaining new receptors (18), encoding antitoxins against bacterial LsoA toxin (19), or encoding anti-CRISPRs proteins (20). Engineered phages have shown promise in combating MDR bacterial infections (8, 18); however, genetic manipulations in phage sequences remain practically challenging, often requiring inefficient, multi-step processes that are time-intensive (21). It has been proposed to use the natural evolvability of phages to counter resistance, termed “phage training,” thereby enhancing their efficacy in suppressing bacterial growth and delaying resistance emergence over time (22). This concept capitalizes on the potential of harnessing phage adaptability as a sustainable strategy to optimize phage therapy outcomes against evolving AMR threats (23).

One important goal of this study was to unravel the genetic mechanisms driving co-evolution dynamics between bacteria and their phages. This puzzling question was addressed by comprehensive sequencing of both evolved bacteria and phages, revealing distinct suites of gene mutations and transcriptional changes. Notably, we characterized a total of 86 evolved bacterial strains by testing their antibiotic susceptibility and *in-vivo* virulence testing in the *Galleria mellonella* model. Our findings demonstrated that a majority of evolved clones exhibited restored antibiotic susceptibility and reduced virulence. In contrast, a significant proportion of evolved phages (60%, *n* = 12/20) showed enhanced bactericidal potency and delayed emergence of resistance. This comprehensive analysis provided valuable insights into the molecular mechanisms governing the co-evolution of bacteria and phages, shedding light on the efficacy of an adaptive training strategy in achieving robust therapeutic outcomes.

## RESULTS

### Evolutionary dynamics between CRKp strain Kp2092 and its lytic phage

**Fig 1:**
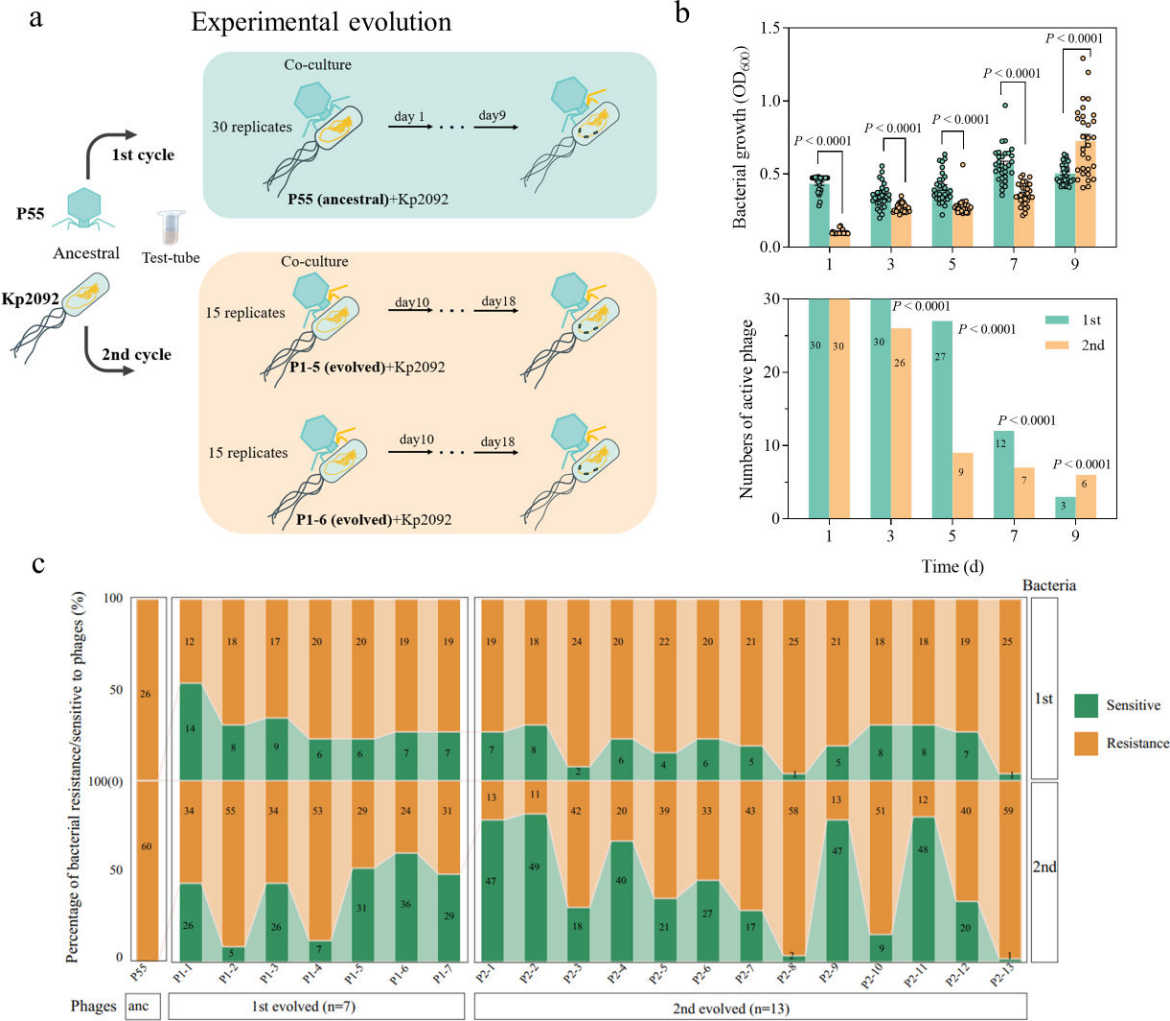
Evolutionary dynamics of phages and bacteria. (**a**) Overview of the co-evolution experiments. Initially, ancestral phage P55 was cocultured with parental strain Kp2092 for 9 days (first cycle). Subsequently, evolved phages P1-5 and P1-6 were isolated and cocultured with parental strain Kp2092 for additional 9 days (second cycle). Phenotypic and genomic characteristics were assessed for both evolved phages and bacteria. (**b**) Bacterial population densities (top) and the number of active phages during two cycles of coculture (*n* = 30 replicates, bottom). (**c**) Percentage of phage sensitive (green) and resistance (orange) strains among evolved bacteria was determined based on spot assays (*n* = 26 from first cycle, *n* = 60 from second cycle). Evolved phages from the first cycle were denoted as P1-, while those from the second cycle were denoted as P2-. Raw data for phage spot assays was also available in source Data S1.

To investigate the evolutionary dynamics between bacteria and their lytic phage, we propagated the CRKp strain Kp2092 with either the ancestral phage P55 or evolved phages over a period of 18 days (Fig. 1a). Strain Kp2092 isolated from sputum belongs to sequence type ST11, encoding multidrug resistance determinants including *bla*_CTX-M-65_, *bla*_KPC-2_, *bla*_SHV-182_, and *fosA6* (24) (the minimum inhibitory concentration [MIC] results are available in the Source Data set S1). Phage P55 belonging to the genus *Przondovirus* with a short tail, is a lytic phage with a broad host range, capable of effectively infecting at least 22 multidrug Kp strains (24). Initially, strain Kp2092 was challenged with the parental phage and serially passaged until phage extinction in all replicate populations (*n* = 30, first cycle), as we have described previously (25). Subsequently, two evolved phages P1-5 and P1-6, isolated from the first cycle, were co-incubated with Kp2092 for another nine-day serial passage (*n* = 15 per phage, second cycle). Bacterial densities with either ancestral or evolved phage P55 steadily increased over a period of nine days (Fig. 1b-top, two-way ANOVA with Sidak’s multiple comparison tests, *P* < 0.0001), indicating the rapid emergence of bacterial resistance. Compared to the ancestral phage group (first cycle), bacterial densities with evolved phages (second cycle) were significantly decreased (two-way ANOVA with Sidak’s multiple comparison tests, *P* < 0.0001, Fig. 1b-top), demonstrating that evolved phages enhanced their ability to suppress bacterial growth. In contrast, the number of active phages significantly decreased, particularly during the second cycle of co-evolution (two-way ANOVA with Sidak’s multiple comparison tests, *P* < 0.0001, Fig. 1b-bottom).

To characterize the evolved phages and bacterial clones, a total of 20 evolved phages and 86 evolved strains were isolated (Table S1). The phage sensitivity of the evolved clones was assessed by spot assays against the ancestral phage P55. As expected, all evolved bacterial isolates were resistant to phage P55 (Fig. 1c; Fig. S1 and S2). Notably, it was challenging for bacteria to develop resistance to evolved phages, especially those from the second cycle (Fig. 1c). For instance, when evolved strains were challenged against newly evolved phages (*n* = 20), only a subset of evolved strains from the first cycle remained sensitive to evolved phages (3.8–30.8%), while phages isolated from the second cycle (e.g., P2-1, P2-2, P2-9, and P2-11), displayed stronger suppression against the majority of evolved strains (66.7–81.7%). The above results illustrate the rapid “arms race” evolution that occurred between phages and their hosts, highlighting the enhanced infectivity of evolved phages against their bacterial hosts.

### Evolution of bacterial phage resistance and its clinical implications

**Fig 2:**
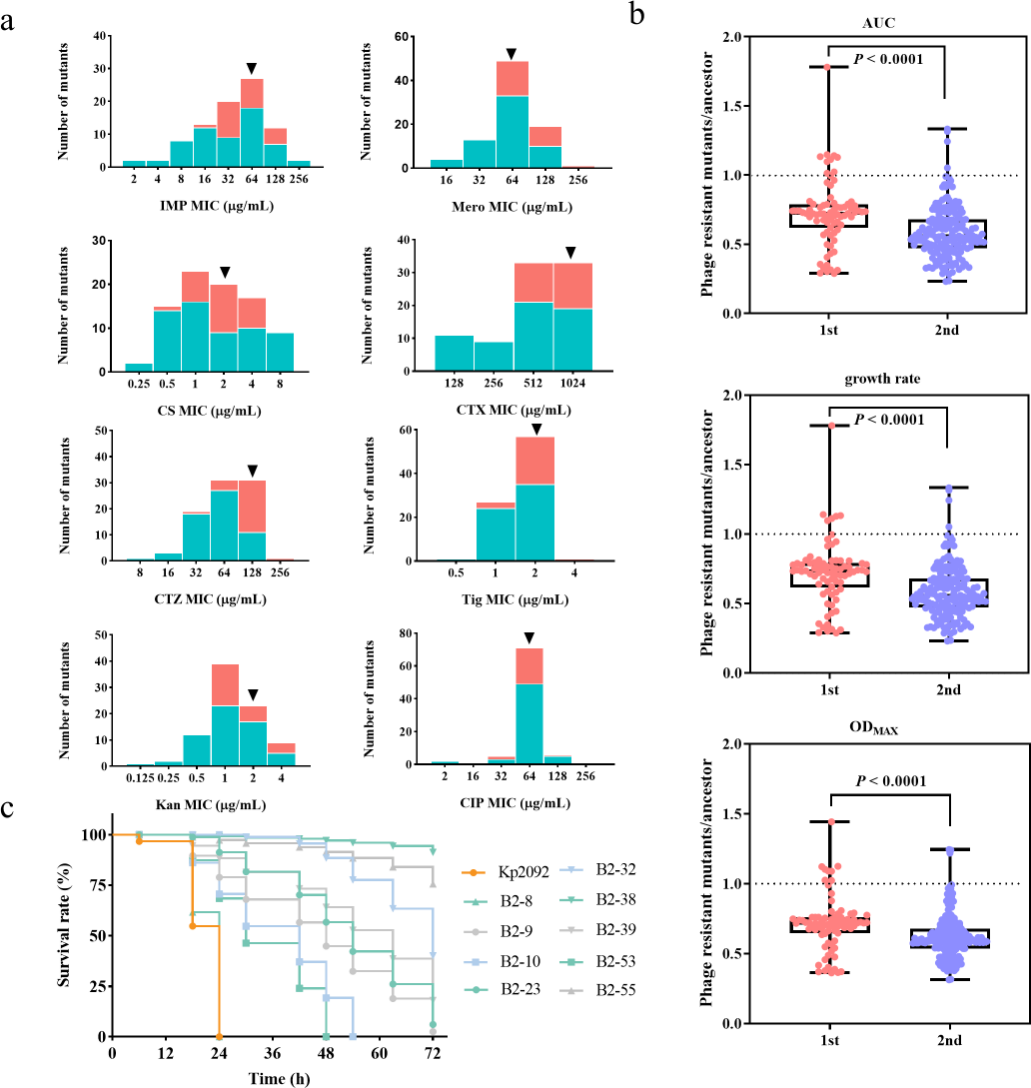
Phenotypic characteristics of evolved bacterial clones. (**a**) Minimum inhibitory concentrations (MICs) of 86 putative evolved isolates to eight clinically important antibiotics: imipenem (IMP), meropenem (Mero), colistin (CS), cefotaxime (CTX), ceftazidime (CTZ), tigecycline (Tig), kanamycin (KAN), and ciprofloxacin (CIP). Evolved isolates from the first cycle are depicted in red, while those from the second cycle are showed in teal. The black triangle indicates the MIC values for the ancestral Kp2092. Exact MIC values for all evolved strains were also available in the source Data S1. (**b**) Assessment of three bacterial growth indicators (area under the curve [AUC], growth rate, and OD_600max_) to evaluate the fitness and competitiveness of evolved bacteria compared to the ancestral strain Kp2092. “1st” denoted evolved bacteria from first cycle, and “2nd” indicated bacteria from second cycle. (**c**) Survival curves of *Galleria mellonella* larvae inoculated with nine evolved bacteria and ancestral strain Kp2092. Kaplan-Meier plots showing the percentage survival of *G. mellonella* larvae over 72 h post-infection. Each experiment was performed in triplicate with 10 animals per treatment per replicate. Statistical analysis was conducted using Survival Mantel-Cox test in GraphPad Prism 8.3.0.

To assess the consequence of evolving phage resistance on clinical outcomes, we examined antibiotic susceptibility, bacterial competitiveness, and *in-vivo* pathogenicity. Compared to the parental strain Kp2092, most evolved bacteria had become re-sensitized to several clinically important antibiotics, including imipenem, meropenem, colistin, tigecycline, and ciprofloxacin (Fig. 2a). This phenomenon aligns with previous findings suggesting that phage resistance evolution is able to lead to increased antibiotic susceptibility in *A. baumannii* (26). Specifically, the average resistance level of evolved strains from the second cycle was generally lower than that from first evolution cycle (Fig. 2a), suggesting the ongoing evolutionary “arms race” between phages and their hosts. Furthermore, bacterial fitness was evaluated through three bacterial growth parameters, area under the curve (AUC), growth rate, and OD_600max_ using the Growthcurver R package (v.4.0.3). The majority of evolved strains displayed reduced fitness compared to Kp2092 (Fig. 2b), while evolved strains isolated from the second cycle exhibited greater fitness burden than those from the first cycle (unpaired *t* test, two-tailed, *P* < 0.0001), indicating the evolved phage was able to effectively reduce pathogenic bacterial load and potentially mitigate infection severity. Using the *G. mellonella* infection model, we assessed the virulence of the evolved strains compared to Kp2092. With the exception of strain B2-8, all evolved clones displayed markedly attenuated virulence (Survival Mantel-Cox test, *P* < 0.0001, Fig. 2c). In particular, strain B2-38, B2-55 only caused 3% and 10% mortality at 72 h, respectively, however, all larvae exposed to Kp2092 died within 24 h (Fig. 2c, *P* < 0.0001, Survival Mantel-Cox test). These results indicate that phage-resistant mutants displayed pleiotropic effects, including increased antibiotic sensitivity, reduced bacterial virulence and compromised competitiveness, potentially offering clinical advantages in interventions against pathogenic MDR infections.

### Genetic mechanisms of phage evolution for enhanced bacterial killing potency

**Fig 3:**
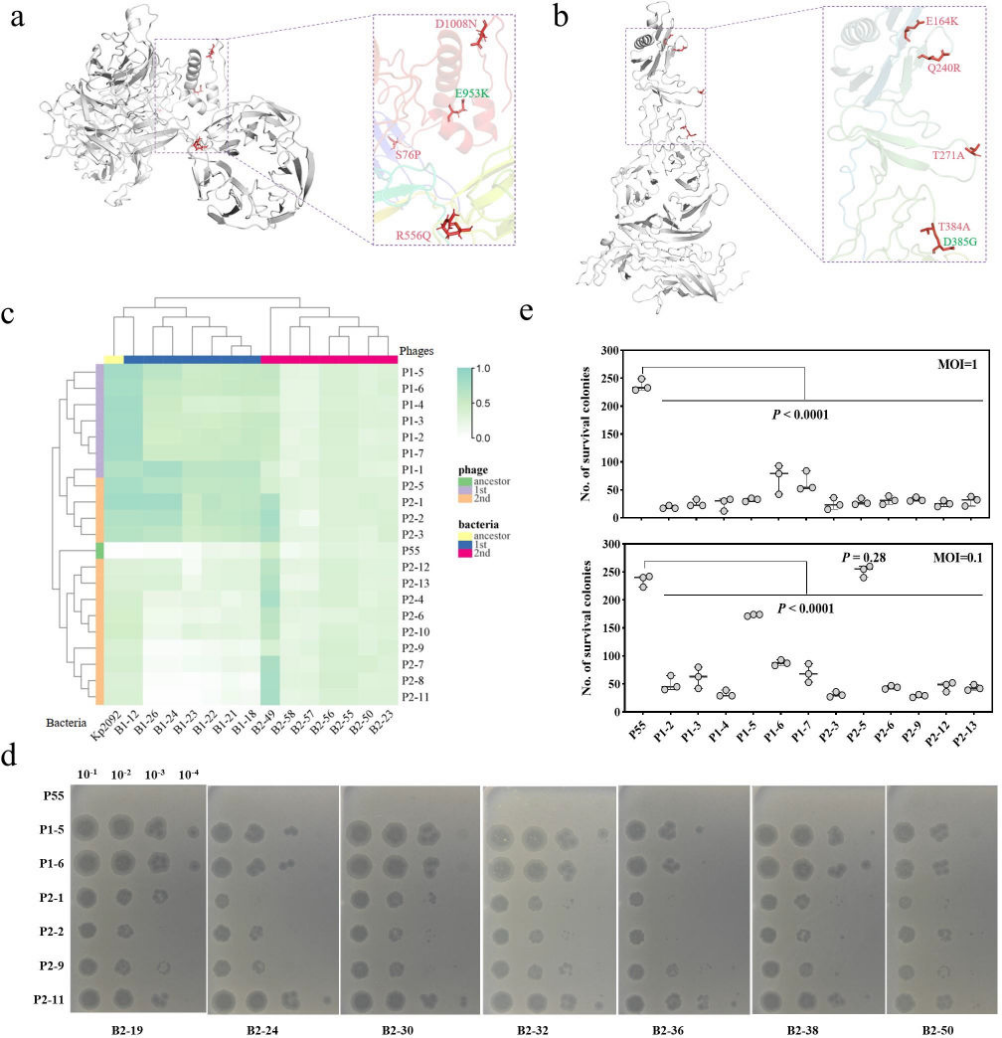
Genetic and phenotypic changes in evolved phages. (**a and b**) Predicted structures of tail fiber protein Gp17 and phage tail tubular protein Gp12, modeled using the I-TASSER server and SWISS-MODEL, respectively. Mutations identified in first cycle were highlighted in green, while those from second cycle were highlighted in red. (**c**) Heatmap displaying iAUC values, illustrating the potency of evolved phages against ancestral Kp2092 and evolved bacteria based on their growth kinetics. iAUC values were calculated as (iAUC = 1 − AUC_sample_/AUC_control_) and were color-coded in shades of green. Exact values of iAUC were also available in source Data S1. (**d**) Number of surviving colonies against ancestral and evolved phages assessed by a lawn-killing assay, indicating delayed emergence of phage resistance against evolved phages, compared to parental phage P55. Each dot represents an independent biological repeat (*n* = 3), with average values shown as boxplots. (**e**) Phage susceptibility of seven selected evolved strains (B2-19, B2-24, B2-30, B2-32, B2-36, B2-38, and B2-50) tested against parental phage P55 and six evolved phages (P1-5, P1-6, P2-1, P2-2, P2-9, and P2-11).

To elucidate the genetic basis for phage evolution, we sequenced the genomes of six evolved phages with enhanced bacterial killing potency. Consistent mutations were identified in two tail structural genes, gp17 (E953K) and gp12 (D385G), across all six evolved phages (Fig. 3a and b; Table S2). These genes encoded the tail fiber protein and tail tubular protein, respectively, both known to inhibit bacterial growth and biofilm formation (27, 28), which probably contributed to the observed enhanced suppression ability of the evolved phages. Additional mutations were identified in the second evolved phages, such as D1008N, G756D, and R556Q in gp17, Q240R, E164K, and T384A in gp12 (Table S2). These mutations are in line with previous studies highlighting gp12 mutations as being crucial for adapting evolved phages to new host receptors (29, 30). Furthermore, the evolved phages from the second evolution obtained more mutations than those from the first evolution, including mutations in the toxin-antitoxin systems, tail tubular protein gp11, and other hypothetical proteins (Table S2). Collectively, these findings suggest genetic adaptability of phages under selective pressure, potentially enhancing their efficacy in therapeutic application against MDR bacterial infections.

To further confirm the lytic potency of the evolved phages (*n* = 20), growth kinetics assays were performed using iAUC index, as described previously (21). All evolved phages significantly suppressed bacterial growth of parental Kp2092 and 14 randomly selected evolved strains, especially those from the first evolution cycle (Fig. 3c), and spot assay showed that evolved phages displayed clear plaques on evolved bacteria where ancestral phage P55 failed to infect (Fig. 3d), suggesting that the evolved phages effectively delayed the emergence of bacterial resistance. To verify this hypothesis, a total of 12 selected evolved phages were selected for lawn-killing assays (21). Consistently, the evolved phages demonstrated greater bacterial killing potency, leading to significantly fewer bacterial survivors compared to the ancestral phage P55 (one-way ANOVA with Dunn’s multiple comparison tests, *P* < 0.0001, Fig. 3e), with the exception of phage P2-5 with a multiplicity of infection (MOI) of 0.1. In addition, evolved phages also exhibited enhanced efficacy against other CRKp strains such as Kp979 and Kp1426, whereas P55 delayed bacterial resistance emergence by only 8–12 h, all tested evolved phages completely suppressed bacterial growth over 24 h (two-way ANOVA with Dunn’s multiple comparison tests, *P* < 0.0001, Fig. S3). These results demonstrate the superiority of evolved phages against clinical CRKp pathogens, highlighting enhanced potency and delayed emergence of bacterial resistance.

### Genetic mechanisms of bacterial phage resistance

**Fig 4:**
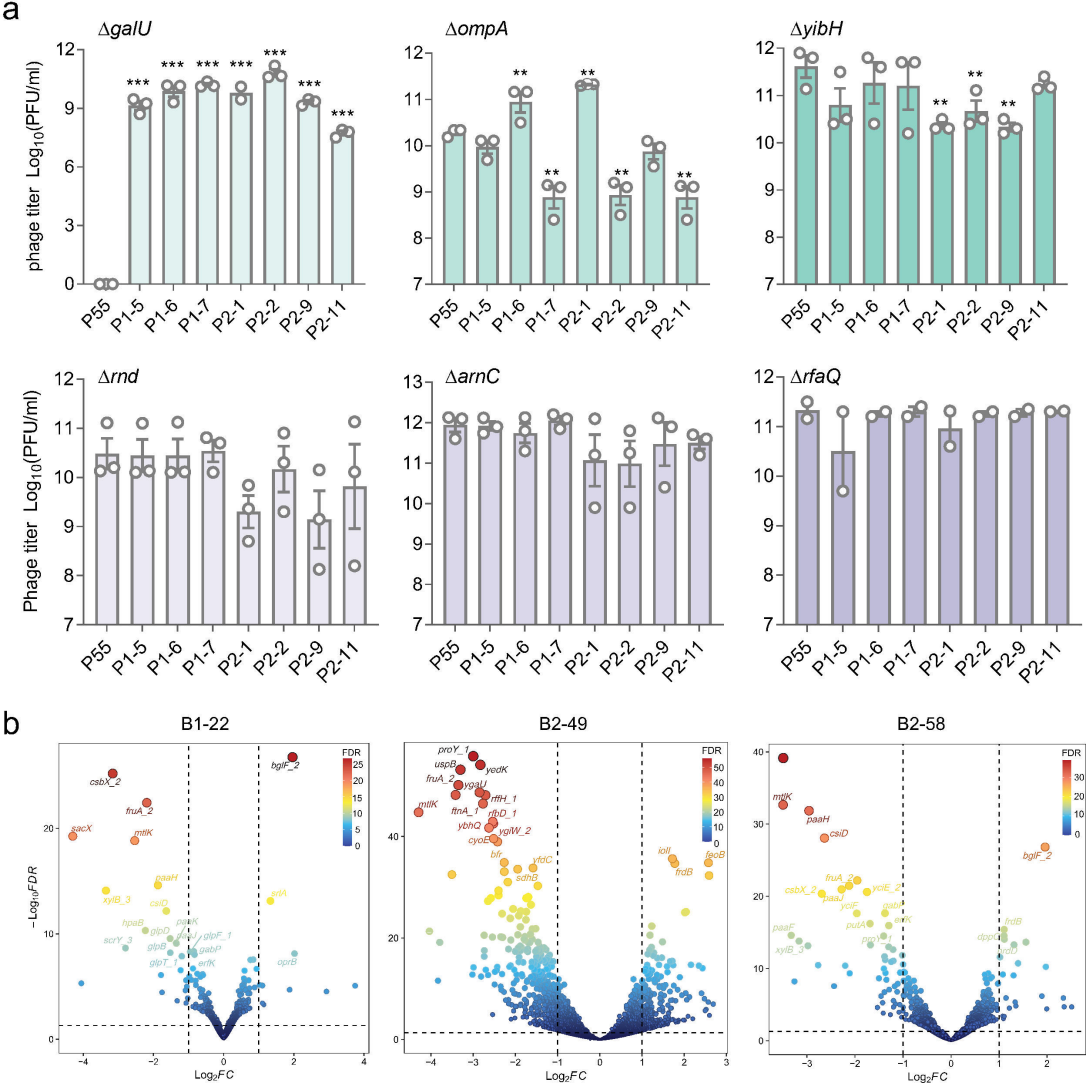
Genetic changes of evolved strains. (**a**) Efficiency of plating (EOP) of ancestral and the respective deletion strains, with phage titers (PFU/mL) shown as dots from three independent biological replicates and averages were indicated as bars. GalU is identified as the primary LPS receptor for ancestral P55 through EOP receptor screening. Data points represent the mean ± SEM from three independent biological repeats (individual dots, *n* = 3). The statistical analysis was performed using an unpaired *t* test to compare mean differences between parental strain and evolved strains (GraphPad Prism 8.3.0). *P < 0.05, **P  < 0.01, and ***P < 0.001; *P* > 0.05 (not significant) was not displayed. (**b**) Volcano plots illustrating changes in the expression level of three most resistant bacteria compared to the ancestral strain Kp2092. The edgeR method was applied for differential expression analysis of RNA-seq data.

To unravel the genetic mechanisms underlying bacterial phage resistance, eight evolved strains were subjected to whole-genome sequencing. Based on core genome SNP analysis, six candidate genes were implicated in phage resistance: membrane protein-encoding genes (*ompA* and *yibH*), three genes encoding functions involved in LPS synthesis (*arnC*, *rfaQ*, and *galU*), and the *rnd* gene responsible for structured RNA processing (Table S3). To further verify the role of these genes in bacterial phage resistance, CRISPR/Cas9-based gene deletions were conducted, followed by plaque efficiency assay against phage P55. The deletion of *galU* abolished plaque formation, whereas its complementation restored phage susceptibility (Fig. 4a; Fig. S4), suggesting *galU* encoded the primary receptor for phage P55 infection. However, the deletion of *galU* did not affect infection by evolved phages (Fig. 4a), indicating that evolved phages may have acquired the ability to use alternative host receptors. Despite the minimal impact on bacterial growth observed upon deletion of *rfaQ*, *galU*, *rnd*, *ompA*, and *yibH* (Fig. S5), a notable decrease in phage plaque formation was observed in several evolved phages, particularly in strains where *ompA* and *yibH* were deleted (Fig. 4a), suggesting these genes play an important role in phage plaque formation.

Furthermore, significant changes in transcriptional profiles were observed, shedding light on the genetic basis underlying bacterial resistance evolution. Comparative analysis between the three most resistant evolved strains (B2-49, B1-22, and B2-58) and the ancestral strain Kp2092, revealed alterations in genes affecting bacterial virulence, antibiotic, and phage susceptibility (Fig. 4b; Table S4). For example, downregulation of *scrY*, analogous to *lamB*, encoding a phage receptor for the phage lambda, was consistent across all three evolved strains (31). Additionally, genes encoding functions involved in LPS synthesis (*lpxL*, *hldD*, *lpxP*, *lapB*, and *pagP*) were downregulated, potentially contributing to resistance against phage infection and virulence loss in evolved strains. In addition, we identified the downregulations of several genes, which may potentially be responsible for the decreased antibiotic resistance (Table S4). For instance, the downregulation of *acrB_2*, a multidrug efflux pump, has been shown to result in reducing bacterial antibiotic resistance and virulence loss (32). These findings highlight the complex genetic adaptations underlying phage resistance, providing insights into mechanisms that could inform strategies for combating MDR pathogens.

## DISCUSSION

Phages possess unique evolutionary advantages over antibiotics, adapting dynamically to counter bacterial resistance. Leveraging these inherent capabilities has emerged as a promising strategy to improve therapeutic efficacy (33, 34). In this study, we examined the co-evolutionary dynamics occurring between clinical CRKp strain and wild-type phages, revealing that the evolved phages showed enhanced lytic ability against both ancestral and evolved bacteria, significantly delaying the occurrence of resistance. Crucially, most evolved strains showed increased sensitivity to clinically important antibiotics and reduced virulence, diminishing their competitive advantage, which ultimately would mitigate the risk of MDR crisis (Fig. 2). Genomic and transcriptional analysis uncovered key genetic mechanisms for both phage and bacterial evolution. Mutations in phage receptors emerged as critical determinants of phage susceptibility, while significant regulation of genes encoding functions involved in LPS synthesis influenced bacterial virulence and phage susceptibility. Our findings demonstrate the robust strategies afforded by bacterial-phage co-evolution to enhance phage therapeutic efficacy by selecting phages with enhanced lytic ability, while concurrently reducing bacterial resistance and virulence.

Our investigation identified primary genetic mutations in the two key phage tail proteins Gp17 and Gp12 (Fig. 3a and b), suggesting a common evolutionary strategy favoring coordinated phage tail assembly and DNA packaging. Specifically, the tail of phage P55 is formed by the tail tubular protein (Gp12) and fiber protein (Gp17), which has been shown to be responsible for phage host recognition and DNA injection (35–37). Mutations in phage tail fibers, particularly those occurring in the C-terminal region, have been associated with increasing their infectivity against resistant strains and broadened host range (38–40). Comparative analysis revealed greater amino acid substitutions in Gp12 and Gp17 among phages from the second cycle (Table S2), correlating with enhanced lytic ability and effective infection of most evolved bacteria (Fig. 1c), which indicated the crucial role of phage tail fiber proteins in infection efficiency. These findings hold significant biomedical implications, particularly when regarding the structural dynamics of phage tails during co-evolution with bacterial hosts. For instance, studies by Huss et al. on T7 phage identified functionally enhanced amino acid substitutions with the tail domain (41), while Ando et al. engineered *Escherichia coli* T7 phage by replacing the whole-tail components, to target new hosts *Yersinia* and *Klebsiella* (42). Yehl et al. engineered T3 phage through directed mutagenesis of tail fiber proteins, yielding “phagebodies” with altered host range and effectively against bacterial resistance both *in vitro* and *in vivo* (18). Therefore, understanding the structure of phage tail proteins and their molecular functions is pivotal in designing strategies to reprogram host range and address resistance problems (43, 44).

Our study revealed that while most evolved bacterial clones retained sensitivity to the evolved phages, 13 strains developed high resistance against all evolved phages (Fig. S2). These strains had evolved several defense strategies against their viral invaders, primarily by modifying phage receptors and modulating bacterial LPS synthesis. These findings emphasize the crucial role of blocking the initial phage adsorption stage to prevent phage infection (45). A mutation in *galU* abolished infection of the ancestral phage P55 but did not affect susceptibility to the evolved phages, indicating phages might have obtained new receptors. This “arms race” evolution has resulted in significant trade-offs for bacterial pathogens, including impaired growth, loss of virulence, and altered antibiotic susceptibility (Fig. 2; Fig. S4), which can be leveraged for clinical intervention (34, 46). Advances in understanding the genetic mechanisms of phage-host interactions have facilitated the development of genetically engineered phages with therapeutic potential. For instance, phages engineered to lack repressor genes have been used successfully to treat *Mycobacterium abscessus* infections (47). Furthermore, phages engineered with CRISPR-Cas bacterial defense systems have shown promise in reducing the spread of AMR genes (48) and lowering *E. coli* burdens in mice models (21). Our study has provided insights into the evolutionary dynamics between clinical pathogens and their specific phages, which merits to further explore a comprehensive range of bacterial hosts for phages and their potential impact in combating AMR.

In conclusion, our evolutionary study highlights the arms race evolution between bacteria and phages, where phage tail proteins were shown to play a critical role in enhancing phage infection efficacy. Evolved phages exhibited promising potential as anti-virulence agents capable of targeting clinical CRKp bacteria and suppressing the occurrence of phage resistance. Therefore, monitoring the evolution of phage-resistant clinical CRKp pathogens and strategically selecting or engineering evolved phages with anti-defense mechanisms may represent innovative approaches in combatting AMR using phages and phage-antibiotic combinations.

## MATERIALS AND METHODS

### Bacteria, phages, and plasmids

The wild-type phage, host bacteria, plasmid, and primers used in this study are shown in Tables S1 and S5. Except when specifically noted, all strains were cultured in Luria-Bertani (LB) broth (Sigma-Aldrich) supplemented with or without kanamycin (Kan, 100 µg/mL), apramycin (Apr, 30 µg/mL), or rifampicin (Rif, 50 µg/mL). The ancestral *K. pneumoniae* Kp2092 and all evolved strains were grown in LB broth at 37°C with shaking of 180 rpm. Whereas the *E. coli* strains containing pCasKP-apr or pSGKP plasmid were grown in LB broth at 30°C. The ancestral strain *K. Pneumoniae* Kp2092 used for the co-evolution experiment was a clinical isolate from the sputum of a patient. The ancestral phage P55 used for co-evolution experiment was a specific lytic phage for CRKp.

### Co-evolution experiment

The co-evolution experiment was performed by using the CRKp Kp2092 with the broad-host range lytic phage P55 belonging to *Przondovirus* for two cycles (Fig. 1a). For the first cycle, we used the ancestral phage P55 and ancestral strain Kp2092 to conduct the 9-day passage coculture. For the second cycle, we used phages P1-5/P1-6 (evolved phages isolated from the first cycle of co-evolution) and the ancestral strain Kp2092 for another 9-day coculture. For each coculture experiment, we performed 30 independent replicates. Specifically, the tubes containing 2 mL LB medium were inoculated with 10^8^ CFU/mL of Kp2092 and 10^8^ PFU/mL of phage and were propagated at 37°C for 180 rpm. After 24 h, the cocultures were transferred into fresh LB medium as 200-fold dilutions. The cocultures were propagated for 9 days. Each day, the titer of phages and bacteria was determined and the corresponding bacteria and phages were isolated. For bacteria, cocultures were diluted in phosphate-buffered saline solution and plated on LB medium, single clones were picked and restreaked at least three times to obtain pure cultures. For phages, cocultures were centrifuged at 12,000 × rpm for 2 min and filtered at least three times using 0.22 µm filter membranes to extract phage lysates. Then, the lysates were serially diluted to 10^−8^, mixed with the ancestral host strain Kp2092 in soft agar, and poured on LB plates. The single plaques were picked and purified three times using the double-layer agar containing normal LB at the bottom and 0.6% top agar. The isolated bacteria and phages were preserved in 15% glycerol at −80°C.

### Spot assays to determine phage susceptibility

An overnight culture of the tested cells was diluted at a ratio of 1:200 and grown to exponential phase and then was mixed with LB medium and poured out to previously prepared cell-containing plates. It was followed by adding 10 µL of the phage lysates on cell-containing plates and incubating overnight at 37°C. The clarity and transparency of the plaques were observed after 20–24 h. The phage susceptibility of bacterial isolates was scored as 1 or 0 (1 = susceptible, 0 = resistant), if there was any plaque on the medium. We assessed the susceptibility of the ancestral strain Kp2092, first evolved bacteria (*n* = 26), second evolved bacteria (*n* = 60) against the ancestral phage P55, first evolved phages (*n* = 7) and second evolved phages (*n* = 13). For evolved bacteria, each isolate was treated as an individual biological replicate, as the first evolved bacteria for 26 replicates and second evolved bacteria for 60 replicates. Any plaques formed were defined as sensitive, whereas no plaques formation was defined as resistant. The resistance/sensitive ratio of bacteria from each cycle was the number of resistance/sensitive divided by the total number of bacteria from each round.

### Growth kinetics assay

*In vitro* susceptibility of ancestral strain Kp2092 to the evolved phages was evaluated using a growth kinetics assay (21). Briefly, overnight cultures of Kp2092 were diluted in LB medium and ∼10^8^ cells inoculated into wells containing 180 µL of LB with 20 µL of phage lysates (∼10^9^) or 200 µL of LB and incubated at 37°C. OD_600_ was monitored using a SpectraMax iD3 plate reader (Molecular Devices) at 1 h intervals over 24 h. Susceptibility was defined based on the values of iAUC (21). The iAUC was calculated following the formula: iAUC = 1 − AUC_Sample_/AUC_Control_ (21), where AUC_Sample_ is the AUC of the spline created by coculture of tested bacteria and phages, while AUC_Control_ refers to the AUC of the spline created with only the tested bacteria. The potency of the evolved phages against the evolved bacteria was also assessed by a stringent *in vitro* growth kinetics assay and calculated with iAUC.

### Lawn-killing assay

The ability for evolved phages to suppress resistance was quantified using a lawn-killing assay (18, 21). Briefly, 100 µL of 10^6^ CFU/mL cells of Kp2092 was mixed with an equal volume of 10^6^ to 10^5^ PFU/mL phage lysates to achieve an MOI of either 1 or 0.1, then these solutions were mixed with 4 mL soft top agar (~45°C) and immediately spread on LB plates. After the top agar solidified, plates were incubated overnight at 37°C, and the total number of surviving colonies was counted for the evolved or the ancestral phage groups for comparison the next day. Each group was accordingly conducted using three independent biological replicates.

### Comparison of bacterial growth using Growthcurver

Overnight cultures of tested cells were diluted at a ratio of 1:400 using fresh liquid medium and 200 µL of the cell solutions was transferred into well plates. The OD_600_ was monitored using a SpectraMax iD3 microplate reader every 1 h for a total of 24 h. The R package v.4.0.3 (Growthcurver) (49) was used for growth rate and fitness calculations based on the data of the growth curve.

### *Galleria mellonella* virulence assays

Virulence changes of evolved bacteria *in vivo* were measured using the *G. mellonella* model, as described previously (50). In brief, the ancestral Kp2092 strain and nine selected evolved strains were grown to exponential phase (OD_600_ = 0.6) in LB broth and then harvested by centrifugation. The bacterial pellets were serially diluted in sterile saline to achieve a density of approximately 10^7^ to 10^8^ CFU/mL, which was injected into the rear left proleg of the larvae using a 10 µL Hamilton syringe (Shanghai, China). The negative control was inoculated with sterile saline. A group of 10 larvae were randomly selected, and three independent replicates were performed for each strain. The infected larvae were maintained at 37°C incubator and the survival rates were recorded every 6 h for up to 72 h. The survival data were analyzed by Kaplan–Meier survival curves (GraphPad Prism 8.3.0).

### Determination of MICs

MICs of antibiotics for both ancestral and evolved strains were performed using the agar dilution method (51) according to Clinical and Laboratory Standard Institute (CLSI) (51). Briefly, one clone from pure cultures of tested cells was picked and resuspended in 1 mL 0.85% saline solution to make a ~10^5^ CFU/mL bacterial suspension. The sterile multipoint inoculation tool was used to dip into the bacterial suspension and then inoculated on the surface of a Mueller-Hinton (MH) agar plate containing serial dilutions of different antibiotics. Three independent biological replicates for each strain and the blank control without antibiotics were set up. Then the plates were incubated at 37°C for 24 h and subsequently recorded. The minimum concentration of antibiotics in the agar plates with no bacterial growth was defined as the MIC (52).

### Whole-genome sequencing and SNP analysis

Total DNA of both ancestral and evolved bacteria and phages were extracted using TIANAmp Bacteria DNA Kit (TIANGEN, China) and DNeasy Blood & Tissue Kit (QIAGEN, Germany), respectively. Paired-end sequencing was performed using Illumina Novaseq 6000. To obtain accurate and high-quality reference genomes, the parental Kp2092 and P55 were also sequenced using the Oxford nanopore MinION platform. Sequencing data of ancestral Kp2092 and P55 based on Illumina and Nanopore were assembled by unicycle v.0.4.8 (53). The assembled sequence was submitted to NCBI (https://submit.ncbi.nlm.nih.gov/) and Prokka (v.1.14.6) for genome annotation. The genomes of the ancestral strain Kp2092 (GeneBank: CP141801.1 for chromosome, CP141802.1 and CP141803.1 for two plasmids) and the ancestral phage P55 (GenBank: OR387886.1) were used as reference genomes of the evolved bacteria and phages, respectively. For the Illumina sequencing data, software BWA (v0.7.17) (54) and SAMTOOLS were used for mapping based on the reference sequence. Individual SNPs and INDEL of evolved bacteria and phages were detected using SAMTOOLS (v1.7) (55). Furthermore, only variant frequencies of 100% were identified and deemed as reliable SNPs.

### Construction of RBDs homology model

The homology model of tail tubular protein was constructed using SWISS-MODEL (56) (http://swissmodel.expasy.org/). Model quality was estimated based on GMQE and QMEANDisCo global score which give an overall model quality measurement between 0 and 1, with higher numbers indicating higher expected quality. The model was built using the structure of phage tail tubular protein B (PDB ID 7Y1C, https://doi.org/10.2210/pdb7Y1C/pdb) from *Klebsiella* phage Kp9 as a template. The sequence identity and similarity between the model and template were 95.83% and 61%. PyMOL v2.0 was used to visualize the structural models (57) (https://www.pymol.org/citing).

### Gene deletion and complementation

Using the two plasmid-based CRISPR-Cas9 system (58), an array of putative phage receptors was deleted via insertion of a PCR-amplified selection marker (kanamycin resistance) within the repair templates to corresponding genome positions. Briefly, the pCasKP-apr plasmid was first electroporated into Kp2092 to obtain the pCasKP-apr-harboring strain. Following induction by l-arabinose, the pCasKP-apr plasmid-containing cells were harvested and made into competent cells. Then, the repaired templates together with target gene spacer-introduced pSGKP-rif plasmid were transformed into pCasKP-harboring competent cells by electroporation. Transformants were selected on LB agar plates containing 50 mg/L rifampin, 30 mg/L apramycin, and 100 mg/L kanamycin. The successful deletion strains were confirmed by PCR and subsequently confirmed by Sanger sequencing. The two plasmids carrying Cas9/homologous recombination system and sgRNA sequence were eliminated by inoculating the mutants on LB medium containing 5% sucrose and incubating at an elevated temperature of 37°C. For complementation, *galU* and its promoter were amplified from Kp2092 and cloned into a pHSG plasmid using Gibson assembly method (59) with NEBuilder HiFi DNA Assembly Master Mix (NEB, USA). The recombinant plasmid was then electroporated into the Δ*galU* mutant competent cells. The successful complementary strains were confirmed by PCR and subsequently Sanger sequencing.

### Efficiency of plaque formation

To better quantify the infective ability of ancestral and evolved phages on different knockout and complementary strains, the efficiency of plaque formation was determined using a double-layer plaque assay (60) with 1.5% LB agar being used at the bottom, while 0.6% LB agar was used as the top agar. Briefly, ancestral Kp2092, knockout, and complementary strains were incubated overnight at 37°C after being inoculated into fresh LB medium. Lysates of ancestral and evolved phages were serially diluted to 10^−8^ using SM buffer. Then, 100 µL of each series of phage lysates was mixed with 100 µL bacterial liquid before being incubated for 10 min at room temperature. After thoroughly mixing bacteria and phage in 0.6% LB top agar (~50°C), the mixture was plated onto the bottom agar. Plaques were observed after overnight incubation at 37°C and the plaque-forming units (PFU/mL) were calculated by dividing the number of plaques for each tested strain by the dilution factor.

### Transcriptomic sequencing

To better explain the underlying transcriptomic mechanisms for phage resistance and the trades-off traits, three representative evolved strains and the parental strain Kp2092 were chosen for RNA-seq. Three replicates were performed for each strain and the data were represented as mean of three replicates. In brief, overnight cultures of the parental strain Kp2092 and three evolved strains were diluted 1:100-folds into fresh LB liquid medium and incubated at 37°C with shaking at 180 rpm. The mid-log phage cultures were harvested by centrifuging for 2 min at 12,000 rpm and quick-frozen by liquid nitrogen. The total RNA was extracted using the TRIzol method. The rRNA was removed using Epicentre Ribo-Zero rRNA Removal Kit (Epicentre, USA). The RNA libraries were constructed using the NEBNext Ultra II Directional RNA Library Prep Kit for Illumina (New England Biolabs, USA). Sequencing was performed on an Illumina NovaSeq PE150 platform. The genome of Kp2092 was used as a reference genome. Differentially expressed genes were detected using DESeq2 (1.34.0) (61) and filtered as *P* value ≤ 0.05, FDR ≤ 0.05, and FoldChange(FC) ≥2. GO and KEGG enrichment was carried out using clusterProfiler (4.2.2).

### Statistical analysis

Data analysis was performed using GraphPad Prism (8.3.0). Data shown in plots are represented as the mean of at least three replicates ± SEM, and the exact number of independent replicates for each experiment was stated in their respective figure legends. Holm-Sidak or Wilcoxon *t* test analysis (*P* < 0.05) and one/two-way ANOVA analysis (*P* < 0.05) were used to compare differences for different treatments or groups.

## Data Availability

All data generated or analyzed during this study are included in the main text and its supplementary files. The DNA sequencing data for phages and evolved bacterial strains are available in the NCBI Sequence Read Archive (SRA) under accession numbers (BioProject accession no. PRJNA1057770, and BioSample accession nos. SAMN39200898 to SAMN39200905). The RNA-seq data generated in this study have been deposited in NCBI database with accession number (BioProject accession no: PRJNA1040450, and BioSample accession nos. SAMN38259569 to SAMN38259580).

## SUPPLEMENTAL MATERIAL

Supplemental material mbio.02957-24-s0001.docx Tables S1-S5; Fig. S1-S5.

Data set S1 mbio.02957-24-s0002.xlsx Additional raw data for Fig.1c, Fig.2a and Fig.3c.

ASM does not own the copyrights to Supplemental Material that may be linked to, or accessed through, an article. The authors have granted ASM a non-exclusive, world-wide license to publish the Supplemental Material files. Please contact the corresponding author directly for reuse.
