# Supplementary material for: Phage-mediated virulence loss and antimicrobial susceptibility in carbapenem-resistant Klebsiella pneumoniae
Source: mBio. 2024 Dec 23;16(2):e02957-24. doi: 10.1128/mbio.02957-24 (PMC11796411; doi:10.1128/mbio.02957-24)
Supplement: Supplemental material — Tables S1-S5; Fig. S1-S5. [file mbio.02957-24-s0001.docx]

Supplementary Materials for

**Phage-mediated virulence loss and antimicrobial susceptibility in** **carbapenem-resistant *Klebsiella pneumoniae***

Yanshuang Yu^1^, Mengzhu Wang^2^, Liuying Ju^1^, Minchun Li, Mengshi Zhao^2^, Hui Deng^2^, [Christopher Rensing](https://www.researchgate.net/profile/Christopher-Rensing-2)^1^*, Qiu E. Yang^1^*, Shungui Zhou

^1^ College of Resources and Environment, Fujian Agriculture and Forestry University, Fuzhou 350002, China.

^2^ Fujian Key Laboratory of Traditional Chinese Veterinary Medicine and Animal Health, College of Animal Sciences, Fujian Agriculture and Forestry University, Fuzhou 350002, China.

* Correspondence: qiueyang2014@163.com (Q.E.Y.); crensing94@gmail.com (C.R.)

**This PDF file includes:**

Supplementary Tables S1 to S5

Supplementary Figures S1 to S5

Source data Excel file. (separate file)

Source data for Fig. 1c, Fig. 2a and Fig. 3c.

**Table S1. The list of strains and plasmids used in this study**

| **Strain ID** | **Species** | **Antibiotic resistance** | **Origin** | **References** |
| --- | --- | --- | --- | --- |
| **Bacteria** | | | | |
| Kp2092 | *K. Pneumoniae* | Carbapenems^R^ | Patient sputum | This study |
| B1-1 | *K. Pneumoniae* | Carbapenems^R^ | evolved clone (1st cycle) | This study |
| B1-2 | *K. Pneumoniae* | Carbapenems^R^ | evolved clone (1st cycle) | This study |
| B1-3 | *K. Pneumoniae* | Carbapenems^R^ | evolved clone (1st cycle) | This study |
| B1-4 | *K. Pneumoniae* | Carbapenems^R^ | evolved clone (1st cycle) | This study |
| B1-5 | *K. Pneumoniae* | Carbapenems^R^ | evolved clone (1st cycle) | This study |
| B1-6 | *K. Pneumoniae* | Carbapenems^R^ | evolved clone (1st cycle) | This study |
| B1-7 | *K. Pneumoniae* | Carbapenems^R^ | evolved clone (1st cycle) | This study |
| B1-8 | *K. Pneumoniae* | Carbapenems^R^ | evolved clone (1st cycle) | This study |
| B1-9 | *K. Pneumoniae* | Carbapenems^R^ | evolved clone (1st cycle) | This study |
| B1-10 | *K. Pneumoniae* | Carbapenems^R^ | evolved clone (1st cycle) | This study |
| B1-11 | *K. Pneumoniae* | Carbapenems^R^ | evolved clone (1st cycle) | This study |
| B1-12 | *K. Pneumoniae* | Carbapenems^R^ | evolved clone (1st cycle) | This study |
| B1-13 | *K. Pneumoniae* | Carbapenems^R^ | evolved clone (1st cycle) | This study |
| B1-14 | *K. Pneumoniae* | Carbapenems^R^ | evolved clone (1st cycle) | This study |
| B1-15 | *K. Pneumoniae* | Carbapenems^R^ | evolved clone (1st cycle) | This study |
| B1-16 | *K. Pneumoniae* | Carbapenems^R^ | evolved clone (1st cycle) | This study |
| B1-17 | *K. Pneumoniae* | Carbapenems^R^ | evolved clone (1st cycle) | This study |
| B1-18 | *K. Pneumoniae* | Carbapenems^R^ | evolved clone (1st cycle) | This study |
| B1-19 | *K. Pneumoniae* | Carbapenems^R^ | evolved clone (1st cycle) | This study |
| B1-20 | *K. Pneumoniae* | Carbapenems^R^ | evolved clone (1st cycle) | This study |
| B1-21 | *K. Pneumoniae* | Carbapenems^R^ | evolved clone (1st cycle) | This study |
| B1-22 | *K. Pneumoniae* | Carbapenems^R^ | evolved clone (1st cycle) | This study |
| B1-23 | *K. Pneumoniae* | Carbapenems^R^ | evolved clone (1st cycle) | This study |
| B1-24 | *K. Pneumoniae* | Carbapenems^R^ | evolved clone (1st cycle) | This study |
| B1-25 | *K. Pneumoniae* | Carbapenems^R^ | evolved clone (1st cycle) | This study |
| B1-26 | *K. Pneumoniae* | Carbapenems^R^ | evolved clone (1st cycle) | This study |
| B2-1 | *K. Pneumoniae* | Carbapenems^R^ | evolved clone (2nd cycle) | This study |
| B2-2 | *K. Pneumoniae* | Carbapenems^R^ | evolved clone (2nd cycle) | This study |
| B2-3 | *K. Pneumoniae* | Carbapenems^R^ | evolved clone (2nd cycle) | This study |
| B2-4 | *K. Pneumoniae* | Carbapenems^R^ | evolved clone (2nd cycle) | This study |
| B2-5 | *K. Pneumoniae* | Carbapenems^R^ | evolved clone (2nd cycle) | This study |
| B2-6 | *K. Pneumoniae* | Carbapenems^R^ | evolved clone (2nd cycle) | This study |
| B2-7 | *K. Pneumoniae* | Carbapenems^R^ | evolved clone (2nd cycle) | This study |
| B2-8 | *K. Pneumoniae* | Carbapenems^R^ | evolved clone (2nd cycle) | This study |
| B2-9 | *K. Pneumoniae* | Carbapenems^R^ | evolved clone (2nd cycle) | This study |
| B2-10 | *K. Pneumoniae* | Carbapenems^R^ | evolved clone (2nd cycle) | This study |
| B2-11 | *K. Pneumoniae* | Carbapenems^R^ | evolved clone (2nd cycle) | This study |
| B2-12 | *K. Pneumoniae* | Carbapenems^R^ | evolved clone (2nd cycle) | This study |
| B2-13 | *K. Pneumoniae* | Carbapenems^R^ | evolved clone (2nd cycle) | This study |
| B2-14 | *K. Pneumoniae* | Carbapenems^R^ | evolved clone (2nd cycle) | This study |
| B2-15 | *K. Pneumoniae* | Carbapenems^R^ | evolved clone (2nd cycle) | This study |
| B2-16 | *K. Pneumoniae* | Carbapenems^R^ | evolved clone (2nd cycle) | This study |
| B2-17 | *K. Pneumoniae* | Carbapenems^R^ | evolved clone (2nd cycle) | This study |
| B2-18 | *K. Pneumoniae* | Carbapenems^R^ | evolved clone (2nd cycle) | This study |
| B2-19 | *K. Pneumoniae* | Carbapenems^R^ | evolved clone (2nd cycle) | This study |
| B2-20 | *K. Pneumoniae* | Carbapenems^R^ | evolved clone (2nd cycle) | This study |
| B2-21 | *K. Pneumoniae* | Carbapenems^R^ | evolved clone (2nd cycle) | This study |
| B2-22 | *K. Pneumoniae* | Carbapenems^R^ | evolved clone (2nd cycle) | This study |
| B2-23 | *K. Pneumoniae* | Carbapenems^R^ | evolved clone (2nd cycle) | This study |
| B2-24 | *K. Pneumoniae* | Carbapenems^R^ | evolved clone (2nd cycle) | This study |
| B2-25 | *K. Pneumoniae* | Carbapenems^R^ | evolved clone (2nd cycle) | This study |
| B2-26 | *K. Pneumoniae* | Carbapenems^R^ | evolved clone (2nd cycle) | This study |
| B2-27 | *K. Pneumoniae* | Carbapenems^R^ | evolved clone (2nd cycle) | This study |
| B2-28 | *K. Pneumoniae* | Carbapenems^R^ | evolved clone (2nd cycle) | This study |
| B2-29 | *K. Pneumoniae* | Carbapenems^R^ | evolved clone (2nd cycle) | This study |
| B2-30 | *K. Pneumoniae* | Carbapenems^R^ | evolved clone (2nd cycle) | This study |
| B2-31 | *K. Pneumoniae* | Carbapenems^R^ | evolved clone (2nd cycle) | This study |
| B2-32 | *K. Pneumoniae* | Carbapenems^R^ | evolved clone (2nd cycle) | This study |
| B2-33 | *K. Pneumoniae* | Carbapenems^R^ | evolved clone (2nd cycle) | This study |
| B2-34 | *K. Pneumoniae* | Carbapenems^R^ | evolved clone (2nd cycle) | This study |
| B2-35 | *K. Pneumoniae* | Carbapenems^R^ | evolved clone (2nd cycle) | This study |
| B2-36 | *K. Pneumoniae* | Carbapenems^R^ | evolved clone (2nd cycle) | This study |
| B2-37 | *K. Pneumoniae* | Carbapenems^R^ | evolved clone (2nd cycle) | This study |
| B2-38 | *K. Pneumoniae* | Carbapenems^R^ | evolved clone (2nd cycle) | This study |
| B2-39 | *K. Pneumoniae* | Carbapenems^R^ | evolved clone (2nd cycle) | This study |
| B2-40 | *K. Pneumoniae* | Carbapenems^R^ | evolved clone (2nd cycle) | This study |
| B2-41 | *K. Pneumoniae* | Carbapenems^R^ | evolved clone (2nd cycle) | This study |
| B2-42 | *K. Pneumoniae* | Carbapenems^R^ | evolved clone (2nd cycle) | This study |
| B2-43 | *K. Pneumoniae* | Carbapenems^R^ | evolved clone (2nd cycle) | This study |
| B2-44 | *K. Pneumoniae* | Carbapenems^R^ | evolved clone (2nd cycle) | This study |
| B2-45 | *K. Pneumoniae* | Carbapenems^R^ | evolved clone (2nd cycle) | This study |
| B2-46 | *K. Pneumoniae* | Carbapenems^R^ | evolved clone (2nd cycle) | This study |
| B2-47 | *K. Pneumoniae* | Carbapenems^R^ | evolved clone (2nd cycle) | This study |
| B2-48 | *K. Pneumoniae* | Carbapenems^R^ | evolved clone (2nd cycle) | This study |
| B2-49 | *K. Pneumoniae* | Carbapenems^R^ | evolved clone (2nd cycle) | This study |
| B2-50 | *K. Pneumoniae* | Carbapenems^R^ | evolved clone (2nd cycle) | This study |
| B2-51 | *K. Pneumoniae* | Carbapenems^R^ | evolved clone (2nd cycle) | This study |
| B2-52 | *K. Pneumoniae* | Carbapenems^R^ | evolved clone (2nd cycle) | This study |
| B2-53 | *K. Pneumoniae* | Carbapenems^R^ | evolved clone (2nd cycle) | This study |
| B2-54 | *K. Pneumoniae* | Carbapenems^R^ | evolved clone (2nd cycle) | This study |
| B2-55 | *K. Pneumoniae* | Carbapenems^R^ | evolved clone (2nd cycle) | This study |
| B2-56 | *K. Pneumoniae* | Carbapenems^R^ | evolved clone (2nd cycle) | This study |
| B2-57 | *K. Pneumoniae* | Carbapenems^R^ | evolved clone (2nd cycle) | This study |
| B2-58 | *K. Pneumoniae* | Carbapenems^R^ | evolved clone (2nd cycle) | This study |
| B2-59 | *K. Pneumoniae* | Carbapenems^R^ | evolved clone (2nd cycle) | This study |
| B2-60 | *K. Pneumoniae* | Carbapenems^R^ | evolved clone (2nd cycle) | This study |
| Kp979 | *K. Pneumoniae* | Carbapenems^R^ | Drainage fluid | This study |
| Kp1426 | *K. Pneumoniae* | Carbapenems^R^ | Patient blood | This study |
| DH5α | *E. coli* | Amp^R^ | Laboratory strain | NEB, #C2987 |
| **Phages** | | | | |
| P55 | *Przondovirus* |  | Sewage-treatment plant | This study |
| P1-1 | *Przondovirus* |  | evolved clone (1st cycle) | This study |
| P1-2 | *Przondovirus* |  | evolved clone (1st cycle) | This study |
| P1-3 | *Przondovirus* |  | evolved clone (1st cycle) | This study |
| P1-4 | *Przondovirus* |  | evolved clone (1st cycle) | This study |
| P1-5 | *Przondovirus* |  | evolved clone (1st cycle) | This study |
| P1-6 | *Przondovirus* |  | evolved clone (1st cycle) | This study |
| P1-7 | *Przondovirus* |  | evolved clone (1st cycle) | This study |
| P2-1 | *Przondovirus* |  | evolved clone (2nd cycle) | This study |
| P2-2 | *Przondovirus* |  | evolved clone (2nd cycle) | This study |
| P2-3 | *Przondovirus* |  | evolved clone (2nd cycle) | This study |
| P2-4 | *Przondovirus* |  | evolved clone (2nd cycle) | This study |
| P2-5 | *Przondovirus* |  | evolved clone (2nd cycle) | This study |
| P2-6 | *Przondovirus* |  | evolved clone (2nd cycle) | This study |
| P2-8 | *Przondovirus* |  | evolved clone (2nd cycle) | This study |
| P2-9 | *Przondovirus* |  | evolved clone (2nd cycle) | This study |
| P2-10 | *Przondovirus* |  | evolved clone (2nd cycle) | This study |
| P2-11 | *Przondovirus* |  | evolved clone (2nd cycle) | This study |
| P2-12 | *Przondovirus* |  | evolved clone (2nd cycle) | This study |
| P2-13 | *Przondovirus* |  | evolved clone (2nd cycle) | This study |
| **Plasmids** | | | | |
| pCasKP | CRISPR-Cas Plasmid | Apr^R^ | Lab plasmid | Ref^1^ |
| pSGKP | CRISPR-Cas Plasmid | Rif^R^ | Lab plasmid | Ref^1^ |
| pHSG299 | Cloning vector | Kan^R^ | Lab plasmid |  |

**Table S2. The list of genomic mutations identified in evolved phages compared with ancestral phage P55**

| **Mutation** | **Strain ID** | | | | | |
| --- | --- | --- | --- | --- | --- | --- |
|  | **P1-5** | **P1-6** | **P2-1** | **P2-2** | **P2-9** | **P2-11** |
| HP (00001) |  | Intergenic (–/+162)  Intergenic (–/+154) |  | Intergenic (–/+154) |  | Intergenic (–/+154) |
| Tail fiber protein gp17 (00006) | E953K | E953K | D1008N  E953K  R556Q  S76P | D1008A  E953K  G756D | D1008A  E953K  G756D  R556Q | D1008G  E953K |
| Tail tubular protein gp12 (00011) | D385G | D385G | D385G | D385G  Q240R | D385G  E164K | D385G  T384A  T271A |
| Tail tubular protein gp11/HP (00012/13) |  |  |  |  | Intergenic (‑46/+19) |  |
| Portal protein (00016) |  |  |  |  |  | V212I |
| Protein 7.3 (00017) |  |  |  | A45V | A45V |  |
| hp (00019) |  |  | E71D | E71D | E71D |  |
| hp (00024) | E80D | E80D |  |  |  | E80D |
| Inhibitor of toxin/antitoxin system (00027) |  |  |  | Q13K | Q13K |  |
| Nucleotide kinase gp1.7 (00037) |  | G83G |  |  |  |  |
| hp (00042) |  |  |  |  | E68* |  |
| T7 RNA polymerase (00043) |  |  | I174V |  |  |  |
| Protein kinase 0.7 (00044) |  |  | W308R |  |  |  |
| hp (00045) |  |  |  | coding (69/180 nt) |  |  |
| hp/hp (00049/50) |  |  |  |  | Intergenic (‑326/+32) |  |
| hp (00051) |  |  | coding  (31/129 nt) | coding  (31/129 nt) | coding  (31/129 nt) |  |

^*^stop codon, nt: nucleotides. No mutation was indicated as blank. hp: hypothetical protein. (00001-00051): the gene number.

**Table S3. The list of genomic mutations identified in evolved bacteria compared with ancestral host Kp2092**

| **Mutation** | **B1-12** | **B1-22^#^** | **B2-49^#^** | **B2-50** | **B2-55** | **B2-56** | **B2-57** | **B2-58^#^** |
| --- | --- | --- | --- | --- | --- | --- | --- | --- |
| *arnC* | coding (308/990 nt) |  |  |  |  |  | Q70* | S158* |
| *rfaQ* |  |  | coding (567/1077 nt) |  |  |  |  |  |
| *rnd* |  |  |  | Q332P |  |  |  |  |
| *ompA* |  |  |  | Intergenic (+33/+99 nt) | L105R | T56P |  |  |
| *yibH* |  | *381Y |  |  |  |  |  |  |
| *galU* |  |  |  |  | E238G |  |  |  |

^*^stop codon, nt: nucleotides. No mutation was indicated as blank. **^#^**the evolved strain was selected for further RNA-seq analysis.

**Table S4. The list of differentially expressed genes involved in phage resistance identified in evolved bacteria compared with ancestral strain Kp2092.**

| Categary | Name | Description | log_2_FC | | |
| --- | --- | --- | --- | --- | --- |
|  |  |  | B1-22  vsKp2092 | B2-49  vsKp2092 | B2-58  vsKp2092 |
| Phage defence | *nikB* | Nickel transport system permease protein NikB | 1.8781 | 2.5688 | 2.3390 |
|  | *tabA* | Toxin-antitoxin biofilm protein TabA | 0.0795 | 1.1190 | 0.2135 |
|  | *scrY_3* | Sucrose porin | -2.7976 | -2.1613 | -2.4357 |
|  | *sacX* | Negative regulator of SacY activity | -4.2984 | -2.9935 | -2.9801 |
|  | *scrK* | Fructokinase | -1.0503 | -1.4307 | -1.3854 |
| Phage defence and virulence | *msbA_2* | Lipid A export ATP-binding/permease protein MsbA | -0.0908 | 1.1614 | 0.0780 |
|  | *lpxL_1* | Lipid A biosynthesis lauroyltransferase | -0.0240 | -1.1396 | -0.3848 |
|  | *lpxP* | Lipid A biosynthesis palmitoleoyltransferase | 0.0678 | -1.3459 | 0.1075 |
|  | *lapB_2* | Lipopolysaccharide assembly protein B | -0.1882 | -1.4627 | -0.2969 |
|  | *pagP* | Lipid A palmitoyltransferase PagP | 0.0047 | -1.7199 | -0.2154 |
|  | *lpxL_2* | Lipid A biosynthesis lauroyltransferase | -0.0839 | -2.0274 | -0.3655 |
|  | *hldD* | ADP-L-glycero-D-manno-heptose-6-epimerase | 0.1195 | -1.2739 | 0.1780 |
| Antibiotic resistance | *uppP* | Undecaprenyl-diphosphatase | -0.0774 | -2.3030 | -0.0013 |
|  | *cmlA* | Chloramphenicol acetyltransferase 2 | -0.0287 | -1.0736 | 0.0335 |
|  | *sttH* | Streptothricin hydrolase | 0.0390 | -2.0888 | -0.2492 |
|  | *-* | hypothetical protein | 0.0075 | -1.4728 | -0.2158 |
|  | *acrB_2* | Multidrug efflux pump subunit AcrB | -0.0866 | -1.2307 | -0.0690 |

**Table S5. The list of primers used in this study**

| **Primers** | **Sequence**  **(5'-3')** | **Size (bp)** | **Application** |
| --- | --- | --- | --- |
| pSGkp-arnC-N20F1 | GGAAGTTTCTGCACGTCACCGTTTTAGAGCTAGAAATAGCAAGTTAAAATAAGGC | ~4500 | *arnC*-Spacer construction |
| pSGkp-arnC-N20R1 | GGTGACGTGCAGAAACTTCCACTAGTATTATACCTAGGACTGAGCTAGC |  |  |
| pSGkp-rfaQ-N20F1 | GGTATCAGCTGAAGATGCAAGTTTTAGAGCTAGAAATAGCAAGTTAAAATAAGGC | ~4500 | *rfaQ*-Spacer construction |
| pSGkp-rfaQ-N20R1 | TTGCATCTTCAGCTGATACCACTAGTATTATACCTAGGACTGAGCTAGC |  |  |
| pSGkp-rnd-N20F1 | GGAATACTCTTCCACCATCGGTTTTAGAGCTAGAAATAGCAAGTTAAAATAAGGC | ~4500 | *rnd*-Spacer construction |
| pSGkp-rnd-N20R1 | CGATGGTGGAAGAGTATTCCACTAGTATTATACCTAGGACTGAGCTAGC |  |  |
| pSGkp-ompA-N20F1 | TAACCCACTGGTATTCCAGAGTTTTAGAGCTAGAAATAGCAAGTTAAAATAAGGC | ~4500 | *ompA*-Spacer construction |
| pSGkp-ompA-N20R1 | TCTGGAATACCAGTGGGTTAACTAGTATTATACCTAGGACTGAGCTAGC |  |  |
| pSGkp-yibH-N20F1 | ATCAACCATTTGATCGGAGAGTTTTAGAGCTAGAAATAGCAAGTTAAAATAAGGC | ~4500 | *yibH*-Spacer construction |
| pSGkp-yibH-N20R1 | TCTCCGATCAAATGGTTGATACTAGTATTATACCTAGGACTGAGCTAGC |  |  |
| pSGkp-galU-N20F1 | AAAGGCAAGAGCCATGACTGGTTTTAGAGCTAGAAATAGCAAGTTAAAATAAGGC | ~4500 | *galU*-Spacer construction |
| pSGkp-galU-N20R1 | CAGTCATGGCTCTTGCCTTTACTAGTATTATACCTAGGACTGAGCTAGC |  |  |
| Kan-PF | TCTCAACCATCATCGATGAATTG | 937 | PCR for full-length kanamycin resistance gene |
| Kan-R | TTAGAAAAACTCATCGAGCATCA |  |  |
| rfaQ-FF | AGAGTCGCTCGCTGGTGCAGGCC | ~1000 | PCR for forward homology arm of *rfaQ* |
| rfaQ-FR | caattcatcgatgatggttgagaGTAAATTCAGGCTGGCTCTGGTG |  |  |
| rfaQ-BF | tgatgctcgatgagtttttctaaGGCGTCATACTCTGTTCTTCATC | ~1000 | PCR for reverse homology arm of *rfaQ* |
| rfaQ-BR | TGCCAGCGTCATGCCCTTCACCT |  |  |
| galU-FF | ATGGCAAGGATGCCCTGCATA | ~1000 | PCR for forward homology arm of *galU* |
| galU-FR | caattcatcgatgatggttgagaAGCCATTGAAATCTCCTGGACTG |  |  |
| galU-BF | tgatgctcgatgagtttttctaaAAACGGCGGTAGCGAAGTAATC | ~1000 | PCR for reverse homology arm of *galU* |
| galU-BR | GGTATTGTCGCGAAGCTGAAG |  |  |
| arnC-FF | AGGCTGCCGCCGACGAAGGCG | ~1000 | PCR for forward homology arm of *arnC* |
| arnC-FR | caattcatcgatgatggttgagaTTATCGCCACGCGGGATAGACC |  |  |
| arnC-BF | tgatgctcgatgagtttttctaaGACGGCAGCCACGATGCTCAATA | ~1000 | PCR for reverse homology arm of *arnC* |
| arnC-BR | TCCTGACCCTGGCGGCACAGTT |  |  |
| rnd-FF | AAGATCTGGCGTTTCTGCAGTAC | ~1000 | PCR for forward homology arm of *rnd* |
| rnd-FR | caattcatcgatgatggttgagaCTGATAGTTCAAAGCATACTCTC |  |  |
| rnd-BF | tgatgctcgatgagtttttctaaTTCGCGGCCAGGTTCAGACCAT | ~1000 | PCR for reverse homology arm of *rnd* |
| rnd-BR | TTGGCAAGACCACCTCCAGC |  |  |
| ompA-FF | AAGCGTTGCAGCAATCGACAG | ~1000 | PCR for forward homology arm of *ompA* |
| ompA-FR | caattcatcgatgatggttgagaCTGCAATCGCGATAGCTGTCT |  |  |
| ompA-BF | tgatgctcgatgagtttttctaaGGCGGCTTAAGTTATAACCGATA | ~1000 | PCR for reverse homology arm of *ompA* |
| ompA-BR | GTACGCAGCCAAACAGCTGTT |  |  |
| yibH-FF | TGACCACGCTGCTGCGGATCA | ~1000 | PCR for forward homology arm of *yibH* |
| yibH-FR | caattcatcgatgatggttgagaCTATCTCGATCACTAAGCCGG |  |  |
| yibH-BF | tgatgctcgatgagtttttctaaCGTAGGTCAGCAACATTAACGTT | ~1000 | PCR for reverse homology arm of *yihH2* |
| yibH-BR | AAGCTGACGCTGACTACGCATC |  |  |
| arnC-F | GCGGCAAATATCTCGCGTTT | ~1000 | PCR Verification for *arnC* deletion |
| arnC-R | CAGATACCGCGGTCGAAGAA |  |  |
| rfaQ-F | TATGGTGCTTAACCTGGCCG | 1095 | PCR Verification for *rfaQ* deletion |
| rfaQ-R | GTTCGTCGGTGTTGGTGTTG |  |  |
| rnd-F | TGCATTTAACCTGATGCCGC | ~1000 | PCR Verification for *rnd* deletion |
| rnd-R | GCTAATCAGCGTTTTGCCGT |  |  |
| ompA-F | TCACGACACCGGTTTCTACG | 1289 | PCR Verification for *ompA* deletion |
| ompA-R | TCGGTGTAGCCCAGAACAAC |  |  |
| yibH-F | CCGCTCAATAAGTGGACCGT | ~1000 | PCR Verification for *yibH* deletion |
| yibH-R | CGGGATAGCCGGAAACATCA |  |  |
| galU-F | CCAAAAGAAATGCTGCCGCT | 1095 | PCR Verification for *galU* deletion |
| galU-R | GAATTTCATCACCAGCGCCC |  |  |
| p+galU-F(galU) | ggtacccggggatcctctagGCAATTGCGTTTATATCTAAGCAGAC | 1140 | PCR for full-length of *galU* with promoter region |
| p+galU-R(galU) | accatgattacgccaagcttTTACTTCGCTACCGCCGTTTC |  |  |
| pHSG-MCS-F2(galU) | AAGCTTGGCGTAATCATGG | 2300 | PCR for full-length pHSG299 |
| pHSG-MCS-R2(galU) | CTAGAGGATCCCCGGGTAC |  |  |

Supplementary Figure S1-S5


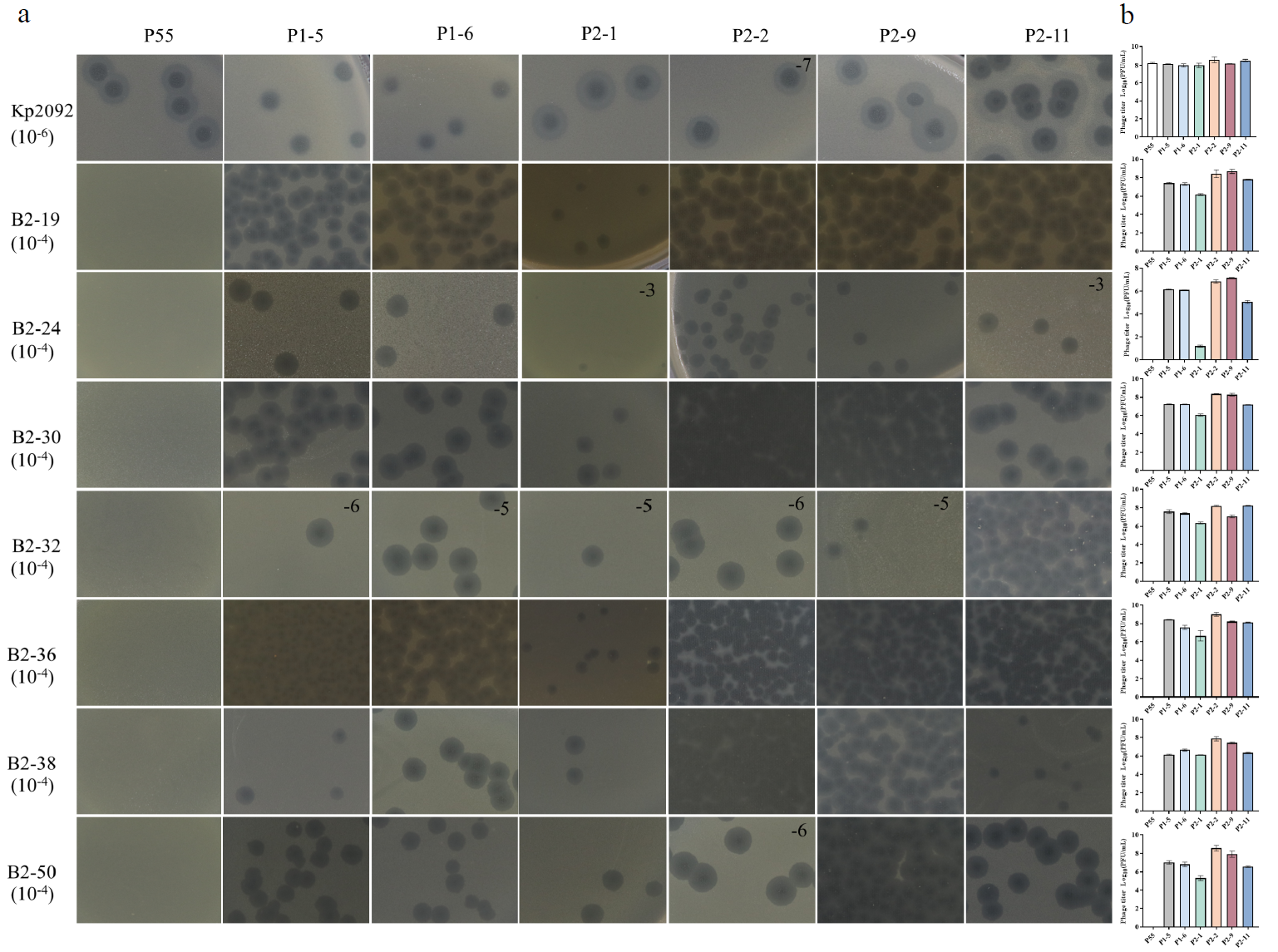


**Fig. S1** (a) Plaque morphologies of six selected evolved phages (P1-5, P1-6, P2-1, P2-2, P2-9 and P2-11) and ancestral phage P55 against seven evolved bacteria (B2-19, B2-24, B2-30, B2-32, B2-36, B2-38 and B2-50) and ancestral host Kp2092. The images were selected under the same dilution factor, the different one was marked with dilution factor upper right corner, the higher numbers of phage plaques were observed in bacterial lawn of evolved bacteria infected by evolved phages, where ancestral phage P55 cannot infect to form plaques, indicating the increased infectivity of evolved phages. (b) The efficiency of plaque formation of six evolved phages and ancestral phage P55 against seven evolved strains and ancestral host Kp2092, determining by double-layer agar plate method. All data is based on three independent replicates (mean ± SEM, n=3).

**Fig. S2** Heat map of all evolved phages (n=26) infecting the ancestral strain Kp2092 and all evolved bacteria (n=60) based on the spot assay results. Variation in phage infectivity are as follows: “0.25” indicates weak phage infection (a few individual plaques); “0.5” medium phage infection (substantial turbidity); “0.75” strong phage infection (mostly clearing); “1” very strong phage infection (complete clearing) or “0” no phage infection (no plaques).

**Fig. S3** Growth kinetics of evolved phages infecting two clinical pathogens *Klebsiella pneumoniae* Kp979 and Kp1426. The data presented represents the mean of three independent experiments with the standard error of the mean (± SEM) indicated.


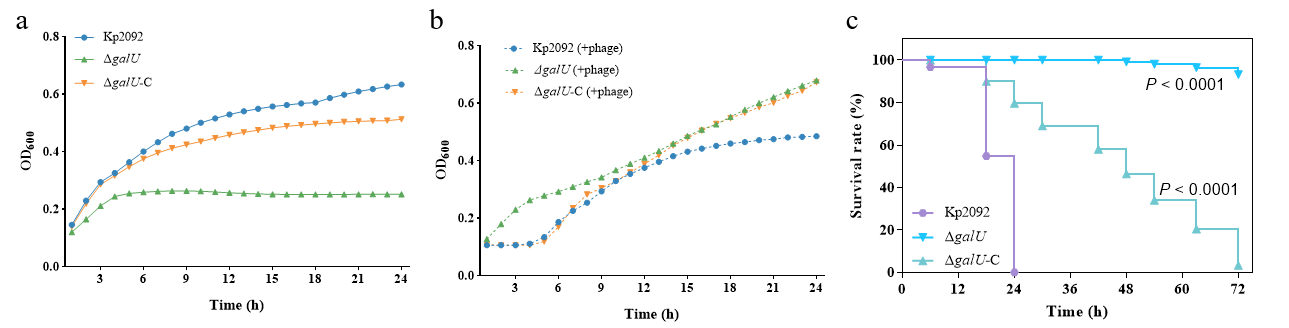


**Fig. S4** Phenotype characterization of ancestral strain Kp2092, *galU* deletion (Δ*galU*) and complementary (Δ*galU*-C) strain. Bacterial kinetic growth curves of three strains on their own (a) or three strains infected with phage P55 (b). *galU* deletion resulted in a substantial decrease in growth rate, whereas complementary of *galU* restored the growth ability. Phage P55 could suppress the growth of Kp2092 and Δ*galU*-C, but not Δ*galU* for 6 h. (c) The physiological effects of *galU* on bacterial virulence were determined by survival rate using *Galleria* *mellonella* infection model. The deletion of *galU* causes substantial decrease on virulence (Survival Mantel-Cox test, *P* < 0.0001) and the complementary of *galU* restored partial virulence (Survival Mantel-Cox test, *P* < 0.0001). Error bars represent the SEM (n=3).


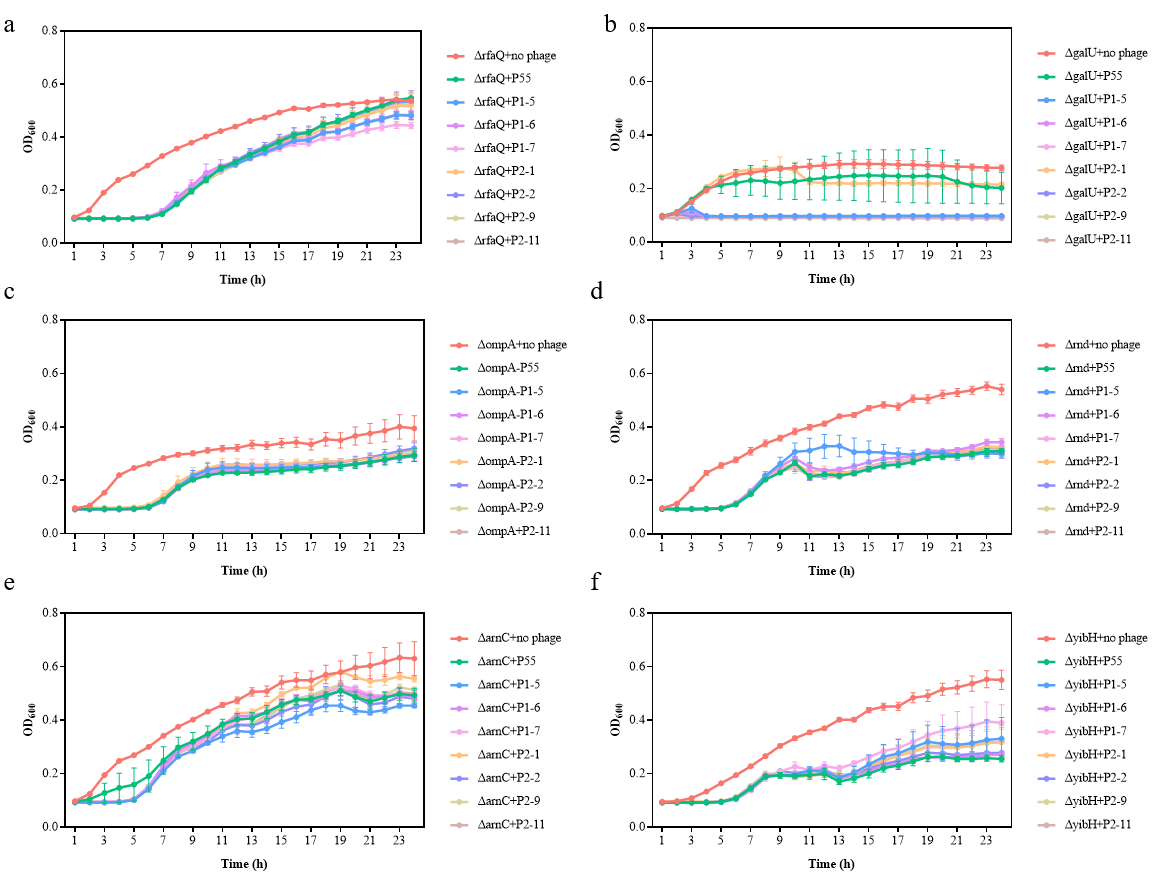


**Fig. S5** The growth kinetics of *rfaQ* (a), *galU* (b), *ompA* (c), *rnd* (d), *arnC* (e) and *yibH* (f) deletion strains genotypes in the absent or presence of 7 evolved phages and 1 ancestral phage P55. Data is based on three independent experiments (mean ± SEM, n=3).

**References**

1. Wang Y*, et al.* (2018) CRISPR-Cas9 and CRISPR-assisted cytidine deaminase enable precise and efficient genome editing in *Klebsiella pneumoniae*. *Appl. Environ. Microbiol.* 84(23): e01834-18
